# Allosteric modulator potentiates **β**_2_AR agonist–promoted bronchoprotection in asthma models

**DOI:** 10.1172/JCI167337

**Published:** 2023-09-15

**Authors:** Seungkirl Ahn, Harm Maarsingh, Julia K.L. Walker, Samuel Liu, Akhil Hegde, Hyeje C. Sumajit, Alem W. Kahsai, Robert J. Lefkowitz

**Affiliations:** 1 Department of Medicine, Duke University School of Medicine, Durham, North Carolina, USA.; 2 Department of Pharmaceutical Sciences, Lloyd L. Gregory School of Pharmacy, Palm Beach Atlantic University, West Palm Beach, Florida, USA.; 3 School of Nursing, Duke University, Durham, North Carolina, USA.; 4 Department of Biochemistry and; 5 Howard Hughes Medical Institute, Duke University School of Medicine, Durham, North Carolina, USA.

**Keywords:** Therapeutics, Asthma, G protein&ndash;coupled receptors, Pharmacology

## Abstract

Asthma is a chronic inflammatory disease associated with episodic airway narrowing. Inhaled β_2_-adrenergic receptor (β_2_AR) agonists (β_2_-agonists) promote — with limited efficacy — bronchodilation in asthma. All β_2_-agonists are canonical orthosteric ligands that bind the same site as endogenous epinephrine. We recently isolated a β_2_AR-selective positive allosteric modulator (PAM), compound-6 (Cmpd-6), which binds outside of the orthosteric site and modulates orthosteric ligand functions. With the emerging therapeutic potential of G-protein coupled receptor allosteric ligands, we investigated the impact of Cmpd-6 on β_2_AR-mediated bronchoprotection. Consistent with our findings using human β_2_ARs, Cmpd-6 allosterically potentiated β_2_-agonist binding to guinea pig β_2_ARs and downstream signaling of β_2_ARs. In contrast, Cmpd-6 had no such effect on murine β_2_ARs, which lack a crucial amino acid in the Cmpd-6 allosteric binding site. Importantly, Cmpd-6 enhanced β_2_ agonist–mediated bronchoprotection against methacholine-induced bronchoconstriction in guinea pig lung slices, but — in line with the binding studies — not in mice. Moreover, Cmpd-6 robustly potentiated β_2_ agonist–mediated bronchoprotection against allergen-induced airway constriction in lung slices obtained from a guinea pig model of allergic asthma. Cmpd-6 similarly enhanced β_2_ agonist–mediated bronchoprotection against methacholine-induced bronchoconstriction in human lung slices. Our results highlight the potential of β_2_AR-selective PAMs in the treatment of airway narrowing in asthma and other obstructive respiratory diseases.

## Graphical abstract

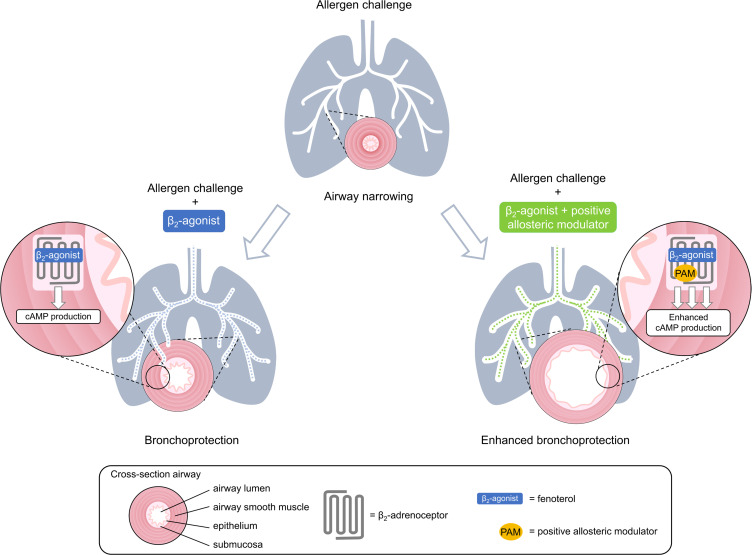

## Introduction

Asthma is a chronic airway disease that affects millions of people worldwide and is the most common chronic disease in children (1). Although asthma is an airway inflammatory disease, it is also characterized by airway hyperresponsiveness and remodeling. The most notable feature of the disease is episodic narrowing of the airways resulting in reversible airflow obstruction known as bronchospasm, asthma attack, or asthma exacerbation. Asthma exacerbation can be provoked by various triggers, including allergens, cold air, exercise, air pollution, and certain chemicals (2, 3). The most common symptoms experienced by asthmatics are those associated with airway narrowing, including wheezing, shortness of breath, chest tightness, and cough. The first line treatment for these symptoms is inhalation of a bronchodilator (4).

The standard of asthma therapy is a combination of inhaled bronchodilators and antiinflammatory drugs. The main bronchodilators are β_2_-adrenergic receptor (β_2_AR) agonists (β_2_-agonists). Inhaled β_2_-agonists are used in the prevention and reversal of airway narrowing by promoting airway smooth muscle relaxation and subsequent improvement of airflow (5). The β_2_AR is a prototypical G-protein coupled receptor (GPCR) that is expressed in airway smooth muscle cells where its activation increases intracellular levels of cyclic AMP via Gαs-mediated activation of adenylyl cyclase (5). The increased levels of cAMP subsequently promote airway smooth muscle relaxation mainly by activation of protein kinase A (PKA) (6, 7), but also via activation of exchange proteins directly activated by cAMP (Epac) (8–10).

β_2_-agonists are fundamental to asthma treatment, providing both prophylaxis (bronchoprotection) and rescue medication (bronchodilation) for many asthmatics. However, several decades of basic, translational, clinical, and epidemiological research has revealed that β_2_-agonists provide suboptimal control for up to 50% of asthmatics (11, 12). Moreover, chronic use of β-agonists has been associated with loss of bronchoprotection, persistent safety concerns, and worsening of asthma control (5). Long-term use of inhaled β_2_-agonists can lead to reduced responsiveness to these medications in individuals with asthma (5, 13) due, in part, to desensitization and downregulation of the β_2_AR (14, 15).

Most drugs targeted to GPCRs bind to the receptor’s canonical orthosteric site, where endogenous ligands bind. All asthma drugs aimed at the β_2_AR are also classic orthosteric ligands (e.g., fenoterol and albuterol [a.k.a. salbutalmol]). Unlike orthosteric ligands, allosteric ligands act at a distance from the orthosteric site and can either positively or negatively modulate the activities of orthosterically acting hormones, neurotransmitters, and drugs (16–18). The therapeutic potential of allosteric drugs lies both in their greater specificity for receptor subtypes, due to reduced evolutionary conservation of allosteric sites compared with orthosteric sites, and their greater safety due to a ceiling effect for their actions. Allosteric effects can be saturated; thus, additional doses of allosteric ligand cause no further effects, hence the ceiling effect (18).

Another potential therapeutic advantage of positive allosteric modulators (PAMs) is that they potentiate the effect of orthosteric agonists, thereby increasing the magnitude of the desired agonist response and/or reducing the dose of the orthosteric agonist required for a desired effect (17). In asthma, positive allosteric modulation of the β_2_AR is an attractive strategy for alleviating asthmatic symptoms given that increased chronic use of β_2_-agonists has been associated with deleterious effects. Moreover, a strong β_2_AR PAM may reduce or obviate the need for β_2_-agonists. Indeed, β_2_AR PAM has the potential to relieve asthmatic airway symptoms during asthma exacerbations through increasing responsiveness of β_2_AR signaling to endogenous levels of adrenaline, which are impaired in asthmatics during asthma exacerbations (19, 20). Thus, PAMs of the β_2_AR might greatly improve the treatment of asthma. However, there have been no such agents available.

We recently isolated a β_2_AR-specific PAM, compound-6 (Cmpd-6), by DNA-encoded library (DEL) screening utilizing the purified receptor occupied by a potent orthosteric agonist and reconstituted into high-density lipoprotein (HDL) particles (a.k.a. nanodiscs) (21). Cmpd-6 displays positive cooperativity with orthosteric agonists, enhancing their binding to the receptor and potentiating agonist-induced downstream cAMP production (21). Physicochemical characteristics of Cmpd-6 can be found in Supplemental Table 1 (supplemental material available online with this article; https://doi.org/10.1172/JC167337DS1) (21, 22). As determined by X-ray crystallography, Cmpd-6 binds to a cytoplasmic cleft outside the central core of the receptor and locks transmembrane (TM) helices 3 and 4 and intracellular loop (ICL) 2 in a conformation only observed in the agonist-activated β_2_AR structures (22). Thus, the action of Cmpd-6 is quite distinct from that of orthosteric β_2_-agonists.

In the current study, we set out to assess whether the functional PAM activity of Cmpd-6 that we previously observed in cellular signaling systems would translate to potentiation of β_2_AR-mediated bronchoprotection in murine and guinea pig models including a model of allergic asthma.

## Results

### Positive allosteric effects of Cmpd-6 on albuterol-mediated responses at the human β_2_AR in functional assays.

**Figure 1:**
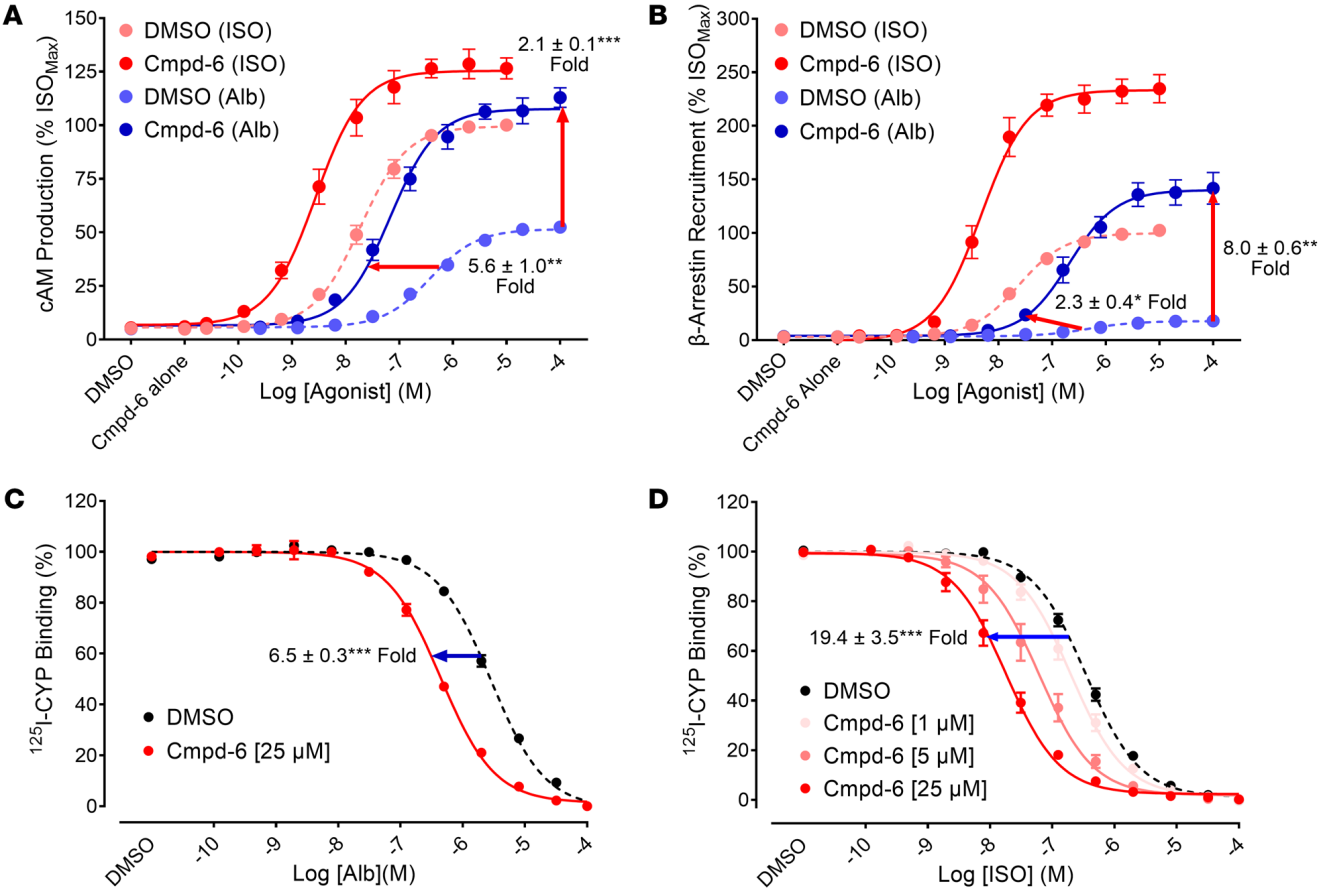
Positive cooperativity of Cmpd-6 with albuterol at the human β_2_AR in functional assays. (**A**) HEK293 cells stably expressing the GloSensor reporter monitoring the cAMP level were pretreated with either 25 μM Cmpd-6 or the control vehicle (DMSO) and stimulated with a serial dilution of either isoproterenol (ISO) or albuterol (Alb). The level of cAMP production induced by the endogenously expressed β_2_AR was determined as described in Methods. (**B**) HEK293T cells expressing all components for the Tango assays monitoring β-arrestin recruitment to the stably expressed β_2_V_2_R as described in Methods were pretreated with Cmpd-6 or DMSO and stimulated with the agonists as described for (**A**). The extent of β-arrestin recruitment was determined as described in Methods. The values in (**A** and **B**) were expressed as percentage of the ISO-stimulated maximal response in the DMSO-treated condition. (**C** and **D**) Isolated membranes from Expi293F cells transiently expressing the human β_2_AR were incubated together with either Cmpd-6 at indicated concentrations or DMSO, a serial dilution of the indicated agonist competitor, Alb (**C**) and ISO (**D**), and 60 pM [^125^I]-cyanopindolol (^125^I-CYP). The reaction was terminated, and ^125^I-CYP bound to the receptor was read as described in Methods. Values were normalized to the percentage of the maximal ^125^I-CYP binding level obtained in each of the Cmpd-6- and DMSO-treated conditions, with data points representing mean ± SEM, obtained from 4 (**A** and **B**) or 5 (**C** and **D**) independent experiments performed in duplicate. The shift of curves was expressed as fold changes in either EC_50_ and B_max_ (**A** and **B**) or IC_50_ (C, D) values between Cmpd-6– and DMSO-treated conditions. Statistical analyses for these shifts in each of the directions were performed using paired 2-tailed Student’s *t* tests. *P* values shown were *P < 0.05, **P < 0.01, ***P < 0.001 compared with the control DMSO-treated condition.

Allosteric modulators often show probe-dependency, displaying preferentially higher cooperativity with a specific orthosteric agonist over others (16–18). Before evaluating the effect of Cmpd-6 on β_2_ agonist–induced airway relaxation, we confirmed cooperativity of Cmpd-6 with albuterol (a.k.a. salbutamol), one of the most commonly used short-acting β_2_-agonists employed for asthma treatment (5). In the presence of Cmpd-6, stimulation of the β_2_AR with albuterol leads to dramatic increases in cAMP production (Figure 1A) and β-arrestin recruitment to the receptor (Figure 1B) in HEK-293 cells, compared with the vehicle (DMSO) control condition. The increases in the maximal response to albuterol are even more robust than those observed with isoproterenol stimulation, as expected, given the partial agonist activity of albuterol. Cmpd-6 also shifts the albuterol dose-response curve to the left in an in vitro competition binding experiment by potentiating the binding affinity of albuterol for the β_2_AR in a concentration-dependent fashion (Figure 1C). However, the shift induced by Cmpd-6 is weaker than with isoproterenol (Figure 1D), which is also consistent with the partial agonist activity of albuterol. Taken together, these data confirm the robust cooperativity between Cmpd-6 and albuterol at the human β_2_AR. This led us to an examination of its allosteric activity in albuterol-induced airway relaxation in an animal model.

### Minimal positive allosteric effects of Cmpd-6 on albuterol-induced bronchodilation in mice.

**Figure 2:**
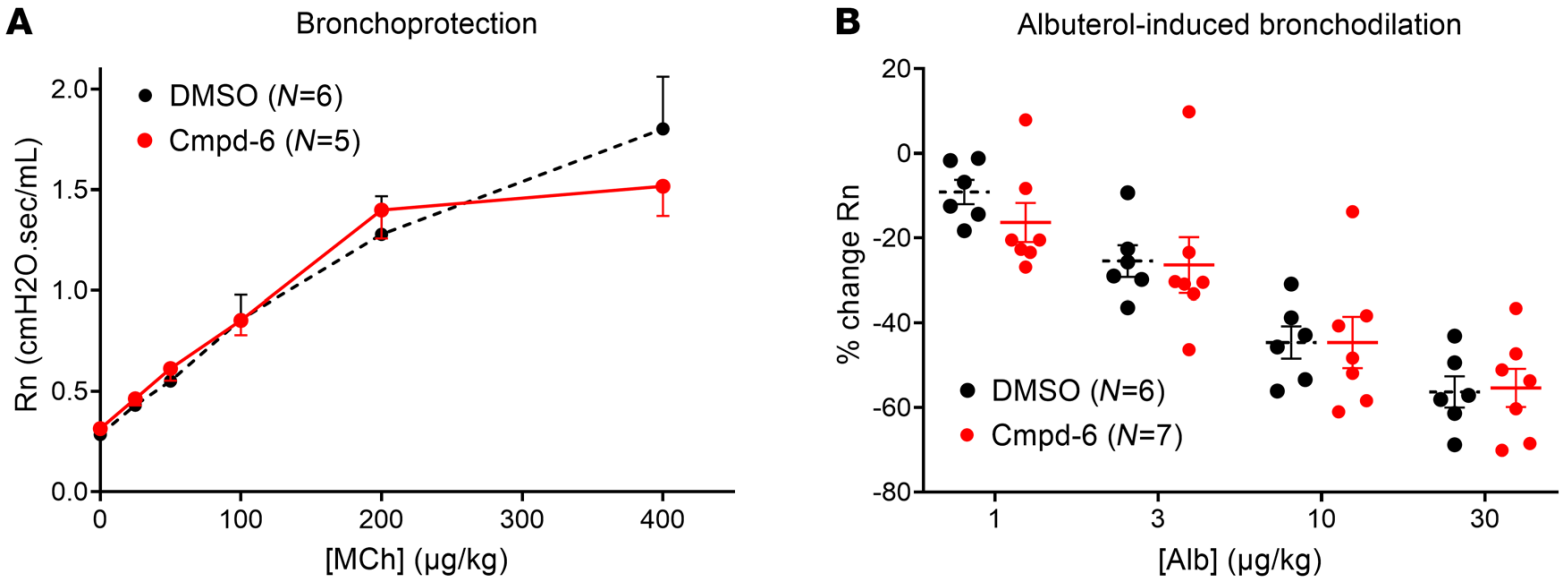
The β_2_AR selective PAM Cmpd-6 does not affect the bronchoprotective effect of β_2_-agonist (albuterol) against methacholine-induced airway constriction in vivo in mice. Effect of Cmpd-6 on (**A**) increasing doses of methacholine-induced (MCh-induced) airway constriction (bronchoprotection protocol) and (**B**) albuterol-mediated (Alb-mediated) bronchodilation of airways preconstricted with methacholine. Anesthetized and skeletal muscle–relaxed C57BL/6J naive mice (*n* = 5–7) were i.v. administered Cmpd-6 (50 mM in 100% DMSO; 10 mg/kg) or equivalent volume of 100% DMSO as vehicle control 10 minutes before the start of the bronchoprotection (**A**) or bronchodilation (**B**) protocol. For the bronchoprotection protocol (**A**), bronchospasm was induced by i.v. administration of methacholine (25, 50, 100, 200, and 400 μg/kg). For the bronchodilation protocol (**B**), methacholine (125 μg/kg) combined with increasing doses of albuterol (1, 3, 10, and 30 μg/kg) was administered i.v. Lung Newtonian resistance (R_n_) was calculated using the forced oscillation technique (flexiVent). Data are represented as mean ± SEM of (**A**) Newtonian resistance (R_n_) or (**B**) percentage (%) change of R_n_ between the average methacholine (125 μg/kg) dose alone and the methacholine plus albuterol combination doses. General Linear Model repeated measures ANOVA with Tukey’s posthoc test was used to determine the differences of airway responsiveness between DMSO- and Cmpd-6–treated conditions.

To examine the effectiveness of Cmpd-6 for enhancing β_2_AR-mediated function in airway smooth muscle, we employed 2 different, but complementary, in vivo assays developed and routinely used in our laboratory (13, 23). To test whether Cmpd-6 offered bronchoprotection in the absence of an exogenous β_2_ agonist, Cmpd-6 was administered before delivery of increasing doses of methacholine, an agonist for muscarinic acetylcholine receptors that induces airway constriction. Secondly, to test the effect of Cmpd-6 on bronchodilation, we preconstricted murine airways with methacholine and administered increasing doses of albuterol in the absence and presence of Cmpd-6. As shown in Figure 2A, airway resistance increased similarly in Cmpd-6–treated and vehicle-treated mice, illustrating no bronchoprotective effect of Cmpd-6. Similarly, there was no enhancing effect of Cmpd-6 on bronchodilation, as evidenced by the nearly identical reduction in airway resistance with increasing doses of albuterol in the absence and presence of Cmpd-6 (Figure 2B). These findings demonstrate that β_2_AR-mediated bronchodilation is not enhanced by Cmpd-6 in mice, which was unexpected based on our in vitro results with the human β_2_AR (Figure 1).

### One of the critical amino acids for Cmpd-6 binding to the β_2_AR, F-133, is not conserved in the murine β_2_AR.

**Figure 3:**
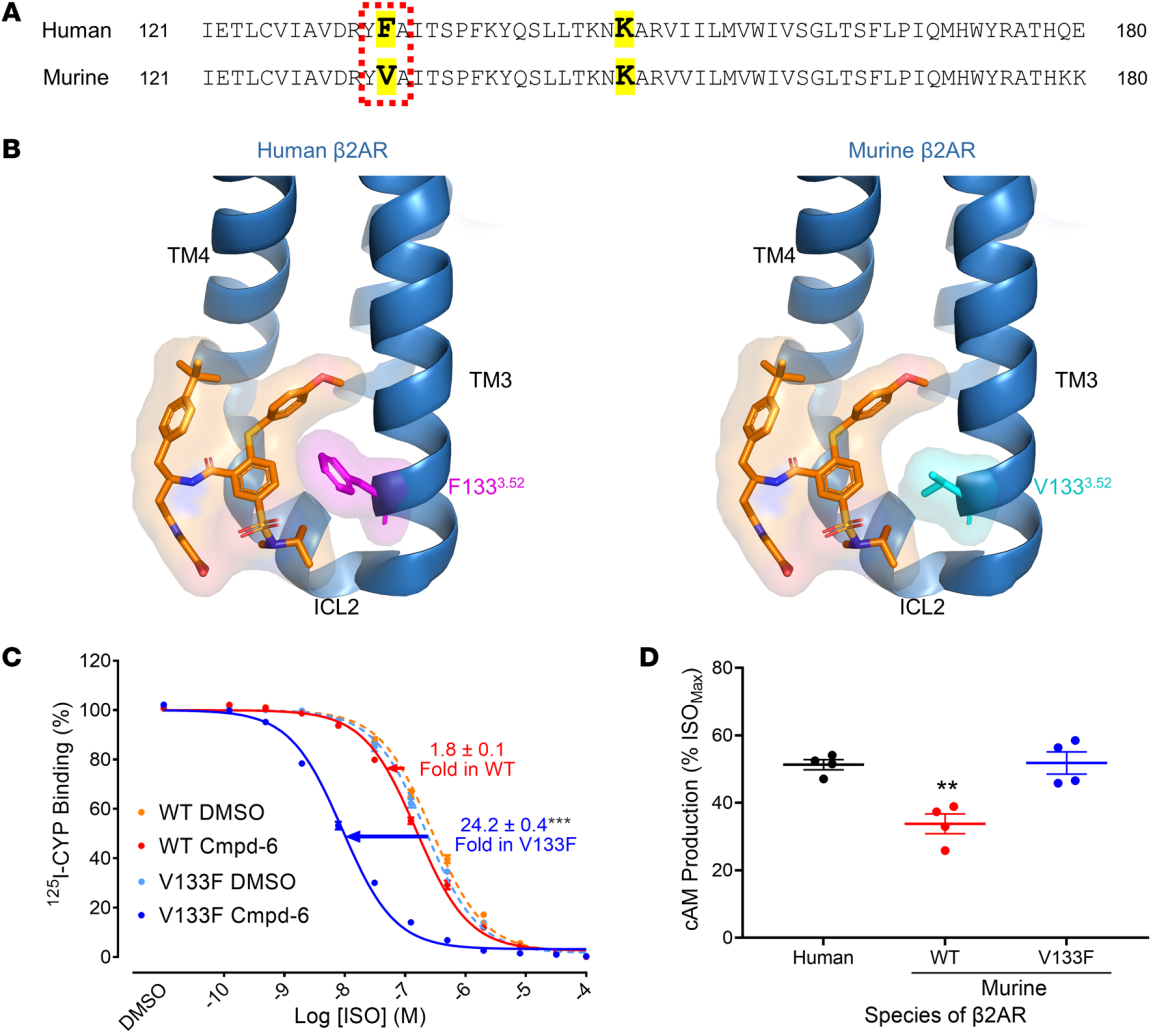
Phenylalanine-133, a critical amino acid for Cmpd-6 binding to the β_2_AR, is valine in the murine β_2_AR. (**A**) The sequence alignment of amino acids composing the Cmpd-6 binding site between the human and murine β_2_ARs. Shaded (yellow) amino acids represent the most essential ones, phenylalanine-133 (F-133), substituted to valine (V) in the murine receptor, and lysine-141 (K-141), for Cmpd-6 binding to the β_2_AR. (**B**) The Cmpd-6 binding site in the human (left) and modeled murine (right) β_2_ARs shows the topographical molecular surface of F-133 (purple) and V-133 (cyan) on the transmembrane-3 (TM-3). Illustrations were created with the previously reported structure PDB-6N48 using the PyMOL program. (**C**) Radioligand competition binding was performed with isolated membranes from 293ExpiF cells transiently expressing either the WT or V133F mutant murine β_2_AR as described in Figure 1, C and D. Curve fits were plotted with data sets obtained from 4 independent experiments done in duplicate. The shift of curves was expressed as fold changes in IC_50_ values between Cmpd-6– and DMSO-treated conditions. Statistical analyses for the shift in each of the WT and mutant receptor were performed using paired 2-tailed Student’s *t* tests. ***P_adj_ < 0.001 compared with the DMSO-treated condition. (**D**) HEK293 cells stably expressing the GloSensor reporter were transiently transfected with one of the human WT, murine WT, and murine V133F mutant β_2_AR. After incubation with Cmpd-6 at 5 μM or DMSO vehicle, the cAMP level was monitored as described in Methods. Values were expressed as percentage of the isoproterenol-stimulated (ISO-stimulated) maximal response obtained as a control for comparable receptor expression in each transfection condition and represent mean ± SEM obtained from 4 independent experiments performed in duplicate. Statistical analyses were performed using 1-way ANOVA, repeated (related) measures with Tukey’s posthoc test. Adjusted **P_adj_ < 0.01.

The recently determined crystal structure of a Cmpd-6 derivative bound to the agonist-activated human β_2_AR indicates that 2 amino acids in the binding site are essential for binding of Cmpd-6: phenylalanine (F)-133 and lysine (K)-142 (22). Since we obtained minimal effects of Cmpd-6 on bronchoprotection or albuterol-induced bronchodilation in our murine model study, we inspected whether these 2 amino acids were conserved in the murine receptor. F-133 and K-142 provide multiple hydrophobic interactions and a hydrogen bond with Cmpd-6, respectively, crucial for Cmpd-6 binding to the human β_2_AR (22). One of these, F-133, is replaced with valine (V) in the murine β_2_AR (Figure 3A). Figure 3B illustrates how substitution of F-133 with V-133 in the Cmpd-6 binding site on the murine β_2_AR hinders Cmpd-6 binding to the murine receptor. The smaller aliphatic side chain of V-133 no longer provides hydrophobic contacts with Cmpd-6 as the larger aromatic ring of F-133 does for Cmpd-6’s binding to its pocket.

In fact, we observed minimal cooperativity of Cmpd-6 with a β_2_-agonist in a competition binding assay with isolated membranes from HEK-293 cells transiently expressing the murine β_2_AR (Figure 3C). On the other hand, we found that mutation of V-133 of the murine β_2_AR to its human counterpart F-133 completely rescued the PAM activity of Cmpd-6 back to the activity observed at the human receptor, as assessed by cooperativity of ligand binding (Figure 3C). We have previously shown that Cmpd-6 behaves like a robust allosteric agonist by further enhancing the high constitutive (basal) cAMP production by the exogenously overexpressed β_2_AR in the absence of orthosteric agonist stimulation (21). This interferes with our ability to assess the positive allosteric effect of Cmpd-6 when the overexpressed β_2_AR is further stimulated with an agonist. Accordingly, we examined the effect of Cmpd-6 on the constitutive activity of overexpressed receptors. The dramatic difference due to a single amino acid change observed in the ligand binding assay (Figure 3C) was precisely recapitulated when we examined the effect of Cmpd-6 on cAMP production induced by the constitutive activity of transiently overexpressed human or murine WT or V133F mutant β_2_ARs (Figure 3D). Mutation of a single residue, V-133 to F-133 of the murine β_2_AR, completely recovered the positive allosteric effect of Cmpd-6 on β_2_AR receptor signaling, thus entirely salvaging the almost absent Cmpd-6 PAM activity observed at the murine WT receptor. These results demonstrate that the minimal effect of Cmpd-6 on airway relaxation in our murine model is likely attributable to its weak PAM activity at the murine β_2_AR due to the crucial single amino acid difference from the human receptor in the Cmpd-6 allosteric binding site.

### F-133 is conserved in the guinea pig β_2_AR and Cmpd-6 serves as a positive allosteric modulator at the guinea pig β_2_AR.

**Figure 4:**
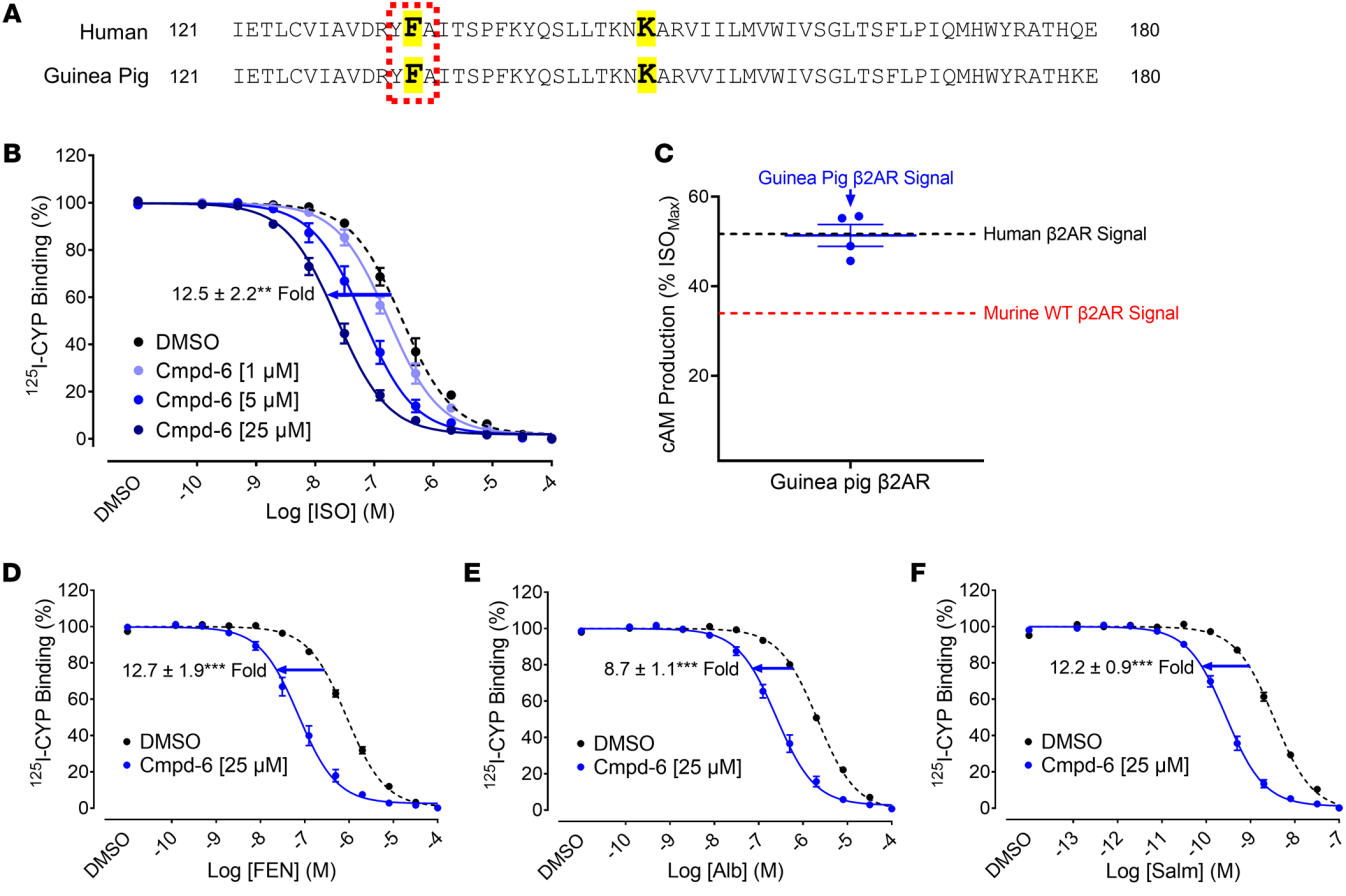
Cmpd-6 serves as a positive allosteric modulator at the guinea pig β_2_AR where Phenylalanine-133 is conserved. (**A**) The sequence alignment of amino acids composing the Cmpd-6 binding site between the human and guinea pig β_2_ARs. Shaded (yellow) amino acids represent the most essential ones, phenylalanine-133 (F-133) and lysine-141 (K-141), for Cmpd-6 binding to the β_2_AR. (**B**) Radioligand competition binding was performed with isolated membranes from 293ExpiF cells transiently expressing the guinea pig β_2_AR as described in Figure 1, C and D. Curve fits were plotted by a 1-site competition binding-log IC_50_ curve fit (GraphPad Prism) with data sets obtained from 5 independent experiments done in duplicate. The shift of curves was expressed as fold changes in IC_50_ values and statistically analyzed using paired 2-tailed Student’s *t* test between the highest concentration of Cmpd-6– and DMSO-treated conditions. **P < 0.01. (**C**) Cmpd-6 was incubated for 20 minutes with HEK293 cells expressing the GloSensor reporter stably and the guinea pig β_2_AR transiently. The extent of cAMP generation was determined and normalized as described for Figure 3D. The value represents mean ± SEM obtained from 4 independent experiments performed in duplicate. The lines indicate the level of Cmpd-6–induced cAMP production driven by overexpression of the human (black) and the murine (red) β_2_AR shown in Figure 3D. (**D**–**F**) Radioligand competition binding was performed as essentially described above for (**B**) with multiple β_2_ agonists as competitors: fenoterol (FEN; **D**), albuterol (Alb; **E**), and salmeterol (Salm; **F**). Curve fits, the expression of the curve shift, and statistical analyses were also generated as described for (**B**) with data sets obtained from 4 independent experiments performed in duplicate. ***P < 0.001.

Since we could not employ the murine system to evaluate the effect of Cmpd-6 on β_2_AR-mediated airway relaxation, we looked for alternative systems to continue our study with Cmpd-6. Fortunately, we discovered that both of the key amino acids, F-133 and L-142 of the human β_2_AR, are conserved in the receptor from guinea pig (Figure 4A), another animal model that is relevant for studying asthma (24, 25). The PAM activity of Cmpd-6 at the guinea pig β_2_AR was tested. Cmpd-6 displayed strong and dose-dependent cooperativity with the β_2_ agonist, isoproterenol, and increased its binding to the receptor, exhibited by the leftward shift of the isoproterenol dose-response curve in a competition binding assay (Figure 4B). We also confirmed Cmpd-6’s PAM activity in cAMP production induced by the constitutive (enhanced basal) activity of the overexpressed guinea pig β_2_AR, which is equivalent to what we observed with the overexpressed human receptor (Figure 4C).

We next examined the cooperativity of Cmpd-6 with other β_2_-agonists utilized to relieve asthmatic symptoms, including albuterol and fenoterol (5) (Figure 4, D and E). At the guinea pig β_2_AR, Cmpd-6 exhibited expected levels of cooperativity with tested β_2_-agonists except salmeterol (Figure 4F). Cmpd-6 showed cooperativity with salmeterol that was stronger than expected given its partial-agonist activity; this may be due to probe dependency. Probe dependency is difficult to predict and results in allosteric ligands that display greater or lesser cooperativity with different orthosteric agonists. Taken together, these findings demonstrate that Cmpd-6 enhances β_2_-agonist binding to, and activation of, the guinea pig β_2_AR.

### Positive allosteric effects of Cmpd-6 on fenoterol-induced bronchoprotection against methacholine-induced airway constriction in guinea pig lung slices.

Since one of the most important therapeutic applications of β_2_-agonists is their effect on β_2_AR-mediated bronchoprotection (5), the positive in vitro findings regarding the action of Cmpd-6 on the guinea pig β_2_AR prompted us to examine its effect on β_2_ agonist–mediated bronchoprotection of intrapulmonary airways in intact guinea pig lung slices using video-assisted microscopy. We first studied whether Cmpd-6 would enhance the bronchoprotection induced by fenoterol — a short-acting full agonist for the β_2_AR — against airway constriction induced by methacholine, an agonist for muscarinic acetylcholine receptors.

**Figure 5:**
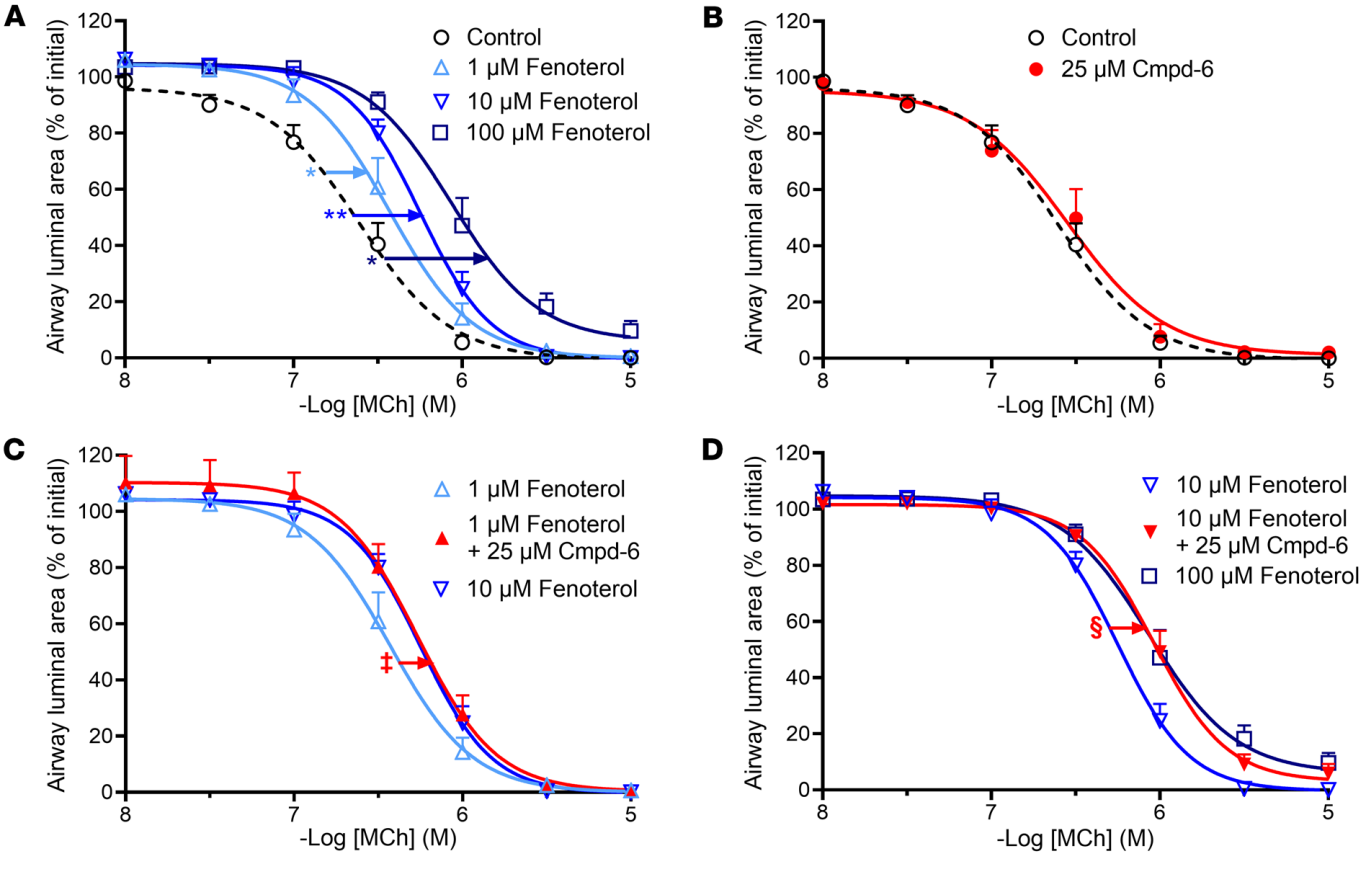
The β_2_AR selective PAM Cmpd-6 enhances the bronchoprotective effect of the β_2_-agonist fenoterol against methacholine-induced airway constrictions in guinea pig lung slices. (**A** and **B**) Lung slices were incubated with 1, 10, or 100 μM fenoterol (**A**) or 25 μM Cmpd-6 (**B**), and airway constriction to increasing concentrations of methacholine (MCh) was determined by measuring the luminal area as a percentage of baseline (**C** and **D**). The effect of 25 μM Cmpd-6 in combination with 1 μM (**C**) or 10 μM (**D**) fenoterol was compared with that at a 10-fold higher fenoterol concentration by itself (10 and 100 μM, respectively). All curve fits were generated using the software program GraphPad Prism. Data are represented as mean ± SEM obtained from 6 guinea pigs. Statistical analyses were performed using a paired 2-tailed Student’s *t* test: *P < 0.05 and **P < 0.01 regarding the shift of the MCh EC_50_ values compared with control; ^‡^*P* < 0.01 and ^§^*P* < 0.01 regarding the additional shift of the MCh EC_50_ values in the presence of Cmpd-6 compared to 1 μM and 10 μM fenoterol, respectively (see also Table 1).

Methacholine induced a concentration-dependent constriction of airways in lung slices obtained from naive guinea pigs with an EC_50_ value of 0.25 ± 0.06 μM (Figure 5A). Whereas treatment with fenoterol (1, 10, and 100 μM) did not affect the maximal airway constriction induced by methacholine, it reduced the sensitivity toward methacholine in a dose-dependent way as indicated by the rightward shift of the methacholine-induced airway constriction response curve and increased EC_50_ values (Figure 5A and Table 1). Treatment with the β_2_AR selective PAM Cmpd-6 (25 μM) by itself did not affect the maximal airway constriction (E_max_) or sensitivity (EC_50_) toward methacholine compared with control (Figure 5B and Table 1).

Importantly, cotreatment with Cmpd-6 enhanced the bronchoprotective effect of 1 μM fenoterol, so that the combined drugs provided the same level of protection as achieved at a 10-fold higher concentration (10 μM) of fenoterol by itself (Figure 5C and Table 1). Similarly, the bronchoprotective effect of 10 μM fenoterol was also enhanced in the presence of Cmpd-6 — again, to the same level of protection as observed at a 10-fold higher concentration (100 μM) of fenoterol alone (Figure 5D and Table 1). These results are consistent with our previous finding that Cmpd-6 potentiates the binding affinity of fenoterol for the guinea pig β_2_AR approximately 10-fold in in vitro binding assays (Figure 4D). Thus, compared with just fenoterol, the same rightward shift in methacholine-induced constriction could be obtained with a 10-fold lower concentration of fenoterol when it was combined with Cmpd-6. The fact that Cmpd-6 is able to augment the responsiveness of the airway smooth muscle to fenoterol by 10-fold suggests that Cmpd-6 may have utility as an adjunctive agent to β_2_-agonists for the treatment of bronchoobstruction in respiratory diseases like asthma.

### Positive allosteric effects of Cmpd-6 on fenoterol-induced bronchoprotection against allergen-induced airway constriction in lung slices from a guinea pig model of asthma.

The therapeutic potential of Cmpd-6 as an adjunctive therapy to treat airway narrowing in asthma was subsequently tested in lung slices obtained from guinea pigs that were actively IgE-sensitized to ovalbumin using Al(OH)_3_ as the adjuvant. Guinea pig models of acute and chronic allergic asthma display characteristics similar to those found in human subjects with asthma, such as airway hyperresponsiveness, eosinophilic inflammation, early and late asthmatic reactions, mucus hypersecretion, and airway remodeling (24). Indeed, guinea pigs are a very relevant species to study pharmacological drug targets in asthma, because the anatomy of the airway, pathophysiology — including mast cell mediators released upon allergen challenge — and receptor pharmacology of guinea pigs are very similar to those of humans (24, 25).

**Figure 6:**
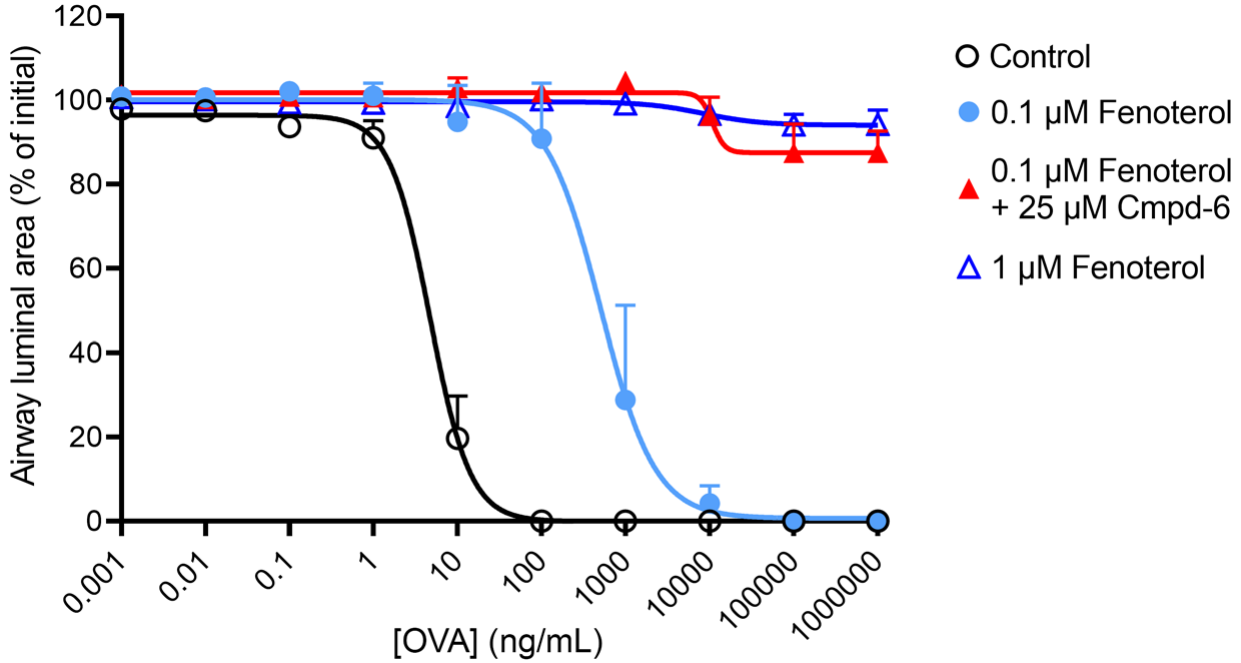
The β_2_AR-selective PAM Cmpd-6 greatly enhances bronchoprotection by the β_2_-agonist fenoterol against allergen-induced airway constrictions in a guinea pig model of allergic asthma. The effect of fenoterol (0.1 and 1 μM) in the absence and presence of Cmpd-6 (25 μM) on ovalbumin-induced (OVA-induced) airway constrictions in lung slices obtained from ovalbumin-sensitized guinea pigs. Lung slices were incubated with increasing concentrations of fenoterol (0.1 and 1 μM) or a combination of fenoterol (0.1 μM) and Cmpd-6 (25 μM), and airway constriction to increasing concentrations of ovalbumin was determined by measuring the luminal area as a percentage of baseline. All curve fits were generated using the software program GraphPad Prism. Data are represented as mean ± SEM of 4–5 guinea pigs (see Table 2 for statistical analyses).

In sensitized guinea pigs, exposure to the allergen (ovalbumin) leads to crosslinking of ovalbumin-specific IgE to the high-affinity IgE receptors, FcεRI, on mast cells, resulting in the release of various mast cell mediators, including histamine (26–28). In line with this, we found that ovalbumin induced a concentration-dependent constriction of airways in lung slices obtained from ovalbumin-sensitized guinea pigs. Ovalbumin induced a full airway constriction with an EC_50_ value of 8.0 ± 2.4 ng/mL (Figure 6 and Table 2). Compared with control, pretreatment of lung slices with 0.1 μM fenoterol induced a 190-fold rightward-shift of ovalbumin-induced airway constrictions without affecting the maximal constriction (Figure 6 and Table 2). Importantly, the bronchoprotective effect of 0.1 μM fenoterol was greatly enhanced in the presence of the β_2_AR PAM Cmpd-6 (25 μM). Compared with treatment with 0.1 μM of fenoterol alone, the cotreatment with Cmpd-6 greatly reduced the maximal constriction from 103.7% ± 2.4% to 19.9% ± 7.7% airway closure – offering almost full protection against allergen-induced airway narrowing. Interestingly, the bronchoprotective effect of 0.1 μM fenoterol plus 25 μM Cmpd-6 was identical to that of a 10-fold higher concentration of fenoterol (1 μM) by itself (Figure 6 and Table 2), consistent with the lung-slice findings observed using methacholine as the contractile agent. These findings show that cotreatment with Cmpd-6 greatly enhanced the bronchoprotective effect of fenoterol against allergen-induced airway constriction.

### Positive allosteric effects of Cmpd-6 on fenoterol-induced bronchoprotection against methacholine-induced airway constriction in human lung slices.

**Figure 7:**
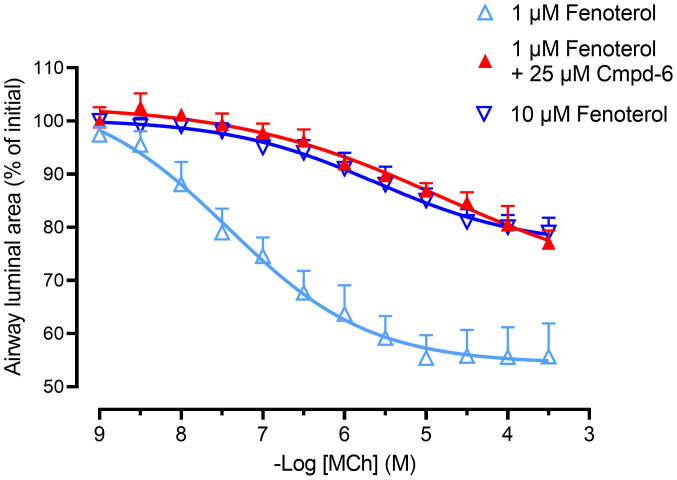
The β_2_AR-selective PAM Cmpd-6 greatly enhances bronchoprotection by the β_2_-agonist fenoterol against methacholine-induced airway constrictions in human lung slices. The effect of fenoterol (1 and 10 μM) in the absence and presence of Cmpd-6 (25 μM) on methacholine-induced (MCh-induced) airway constrictions in human lung slices. Lung slices were incubated with increasing concentrations of fenoterol (1 and 10 μM) or with a combination of fenoterol (1 μM) and Cmpd-6 (25 μM), and airway constriction to increasing concentrations of methacholine was determined by measuring the luminal area as a percentage of baseline. All curve fits were generated using the software program GraphPad Prism. Data are represented as mean ± SEM of 4 human donors (see Table 3 for statistical analyses).

Since asthma is a human disease, it was essential to assess the ability of Cmpd-6 to enhance β_2_ agonist–mediated bronchoprotection in human tissue. We therefore tested the ability of Cmpd-6 to enhance β_2_ agonist–mediated bronchoprotection against methacholine-induced airway narrowing in human lung slices. As one would expect based on classic pharmacologic principles, Figure 7 shows that 10 μM fenoterol offered better protection against methacholine-induced bronchoconstriction than 1 μM fenoterol. (Figure 7 and Table 3). In the presence of 1 μM fenoterol, the maximal methacholine-induced airway constriction was 47.2% ± 5.2% with a pD_2_ value of 6.99 ± 0.10. The bronchoprotection by 10 μM fenoterol was enhanced as shown by lower maximal methacholine-induced constrictions, 21.7% ± 2.2%, and a rightward shift of the pD_2_ value to 5.77 ± 0.33 (Figure 7 and Table 3). In other words, the airway luminal area at the highest methacholine concentration was larger with 10 μM fenoterol (79.5% ± 2.8% open) than with 1 μM fenoterol (55.7% ± 6.2% open). Importantly, Figure 7 shows that when 25 μM Cmpd-6 was added to 1 μM fenoterol, the effectiveness of the β_2_-agonist in preventing methacholine-induced bronchoconstriction was greatly enhanced, reducing the maximal methacholine-induced constriction to 26.5% ± 4.4% and inducing a rightward shift of the pD_2_ value to 5.52 ± 0.24 (Figure 7 and Table 3). Additionally, the bronchoprotective effect of 1 μM fenoterol plus 25 μM Cmpd-6 was indistinguishable from that of 10 μM fenoterol (Figure 7 and Table 3), demonstrating a 10-fold enhancement of the β_2_-agonist responsiveness. The result that Cmpd-6 enhances β_2_ agonist–mediated bronchoprotection in human lung slices is consistent with that observed in guinea pig lung slices. Moreover, the functional relevance of Cmpd-6 in humans that was implied by the results of the human cell in vitro positive cooperativity binding and signaling studies was confirmed by these findings in ex vivo human airways.

## Discussion

Previously, we showed that Cmpd-6 enhances binding of the orthosteric ligand, isoproterenol, at the β_2_AR and potentiates downstream cAMP production (21). Here, we demonstrate the therapeutic potential of Cmpd-6 to enhance airway smooth muscle relaxation in response to β_2_-agonists commonly used in the treatment of asthma. Our in vitro studies using guinea pig β_2_AR show that Cmpd-6 improves agonist binding of fenoterol, albuterol, and salmeterol to the guinea pig β_2_AR and enhances cAMP production. Additionally, the positive allosteric effect of Cmpd-6 on the physiological function of β_2_AR was demonstrated; we showed that Cmpd-6 augmented the fenoterol-induced bronchoprotection against methacholine-induced bronchoconstriction in guinea pig lung slices. In fact, when Cmpd-6 was added, the same bronchoprotective effect could be achieved with a 10-fold lower dose of the β_2_ agonist. To test if the positive allosteric bronchoprotective effect of Cmpd-6 was pathophysiologically relevant, we used lung slices from a guinea pig model of allergic asthma and showed that Cmpd-6 robustly enhanced fenoterol-induced bronchoprotection against allergen-induced airway constriction. Importantly, we demonstrated that the positive allosteric effect of Cmpd-6 on fenoterol-induced bronchoprotection was also present in human tissue, where the combination of Cmpd-6 and fenoterol again offered the same level of protection as that observed with a 10-fold higher dose of the β_2_ agonist. Taken together, these findings demonstrate that the β_2_AR-selective PAM, Cmpd-6, enhances agonist binding to, and signaling of, guinea pig and human β_2_ARs and potentiates β_2_ agonist–mediated bronchoprotection in naive and allergic guinea pig lung slices and human lung slices. These results suggest that β_2_AR PAMs, like Cmpd-6, could have important clinical utility for the treatment of airway narrowing in asthma.

Recently, an increasing number of positive and negative allosteric modulators for GPCRs have been described (16–18); although, to date, only 2 have reached the clinic (29, 30). Rather than directly stimulating or inhibiting biological effects, allosteric modulators exert their effects by altering receptor responsiveness to orthosteric agonists. Allosteric compounds offer a number of potential therapeutic advantages compared to classical orthosteric ligands, including improved safety, reduced off-target effects, and increased efficacy or potency of orthosteric ligands. Drug safety may be improved since the effects of PAMs are saturable and therefore exhibit a ceiling effect. Thus, the risk of overdosing is reduced and is only manifest when an orthosteric agonist is present (17, 18). Off-target effects are reduced by PAMs compared with orthosteric agonists because the former bind with greater specificity among closely related receptor subtypes. Orthosteric binding site regions are highly conserved among receptor subtypes within a receptor family since these subtypes all bind the same endogenous agonist(s). On the other hand, allosteric regions of receptor subtypes are evolutionarily less conserved, and thus allosteric compounds provide greater binding specificity among closely related receptor subtypes.

In asthma, the addition of PAMs like Cmpd-6 could improve the efficacy and reduce unwanted side effects of currently prescribed β_2_-agonists. In terms of efficacy, PAMs for the β_2_AR should reduce the dose of β_2_-agonists needed to obtain clinically relevant outcomes (e.g., bronchoprotection) and enhance the physiological response to currently prescribed doses of β_2_-agonists. PAMs might also potentiate the airway effects of endogenous epinephrine in asthmatics. These scenarios might well result in better clinical outcomes, such as better asthma control and reduced tachyphylaxis for patients that progressively become less responsive to β_2_-agonists.

Adverse drug responses to inhaled β_2_-agonists are dose dependent and in some cases drug specific (31, 32). Despite the development and use of β_2_-agonists in asthma and COPD that display clear selectivity toward β_2_ARs over β_1_ARs, there remains some degree of cross-reactivity, particularly at higher treatment doses. Typical β_1_AR-mediated cardiac effects, such as arrhythmias, increased myocardial oxygen demand, and sudden death are still a concern in asthmatic patients, despite using selective β_2_-agonists. While some of these cardiac events may be β_2_AR-mediated, off-target β_1_AR activation certainly plays a significant role (31, 32). The strong selectivity of Cmpd-6 for the β_2_AR over the closely related β_1_AR would preclude any increase in β_1_AR-mediated side effects associated with its use. The risk for these cardiac side effects may even decrease if β_2_AR-selective PAMs permit a lower effective dose of β_2_-agonist to be prescribed.

Although Cmpd-6 alone, in the absence of fenoterol, did not reduce methacholine-induced airway constriction in guinea pig lung slices, this result does not rule out the possibility that monotherapy with Cmpd-6 could have beneficial effects in asthma. Humans, unlike the isolated lung slices used in our experiments, have circulating levels of epinephrine, the β_2_AR binding and signaling effects of which would be improved by PAMs. The fact that nonselective βAR-blockers are contraindicated in individuals with asthma (33) supports the notion that endogenous epinephrine offers a modicum of bronchodilation in this disease. However, endogenous epinephrine alone is clearly not able to adequately counteract airway narrowing in asthmatics. This may be explained by the observation that levels of catecholamines, including epinephrine, fail to rise normally during an asthmatic exacerbation (19, 20) and in response to exercise in individuals with exercise-induced asthma (34). It is tempting to speculate that PAM-induced enhancement of β_2_AR responsiveness to endogenous epinephrine may protect against the development and/or severity of asthma exacerbations. Thus, a strong β_2_AR-specific PAM may improve asthma control by endogenous epinephrine and thus reduce or obviate the need for long-term use of inhaled β_2_-agonists without increasing the risk for epinephrine-induced adverse effects via other adrenergic receptor subtypes.

The mechanisms of β_2_AR tachyphylaxis in asthma are not well understood and we did not use a model of β_2_AR tachyphylaxis; thus, it is difficult to speculate as to the ability of Cmpd-6 to impact its development and/or progression. Possible molecular mechanisms for functional β_2_AR tachyphylaxis include reduced density of cell surface receptors — such as an imbalance in ratios of receptor production to degradation and/or internalization to recycling (35, 36) — phosphorylation-mediated uncoupling of receptors from downstream Gs/cAMP signaling (37–39), increased activity of endogenous receptor desensitization by β-arrestins (13, 15, 40), some combination of all 3, or an, as yet, undiscovered mechanism (5, 14, 41). Given that Cmpd-6 enhances β_2_ agonist–mediated bronchoprotection while also enhancing agonist-induced β-arrestin recruitment to the β_2_AR, we would not anticipate that Cmpd-6 would reduce receptor desensitization. However, additional study of receptor tachyphylaxis mechanisms is needed and doing so in the context of PAMs is warranted.

PAMs like Cmpd-6 would likely be more effective in treating asthma if they were Gs-biased, since β-arrestin signaling is proasthmatic in murine models and β-arrestin binding leads to receptor desensitization. β-arrestin2 is required for the development and perpetuation of the asthma phenotype in mice (23, 42). Murine lung expression of β-arrestin2 is upregulated by allergen sensitization and challenge (13). β-arrestin2 mediates agonist-specific β_2_AR desensitization in airway smooth muscle (15, 40), and knockout of β arrestins prevents β_2_-agonist–induced functional tachyphylaxis (15, 23, 40, 42). Thus, future efforts to develop Cmpd-6 analogs that are Gs-biased would be worthwhile.

In human asthma, the hallmark signs and symptoms, including airway constriction, are caused by release of histamine and leukotrienes from mast cells and other inflammatory cells (43) as well as by release of acetylcholine from airway neurons and from nonneuronal sources, such as airway epithelium and inflammatory cells (44). Allergen-induced airway constriction in our guinea pig model is mainly caused by histamine released from activated mast cells (27, 28). Allergens cause mast cell activation and degranulation via allergen-specific IgE crosslinking with the high-affinity IgE receptor, FcεRI (26). Our observation that fenoterol provided better bronchoprotection against ovalbumin-induced, compared with methacholine-induced, airway constriction could be explained by the findings that fenoterol reduces immediate antigen-induced histamine release from human (45–47) and guinea pig (48) mast cells and is better at counteracting tracheal contractions induced by histamine than by methacholine (49, 50). It is important to note that, despite the more profound bronchoprotective effect against ovalbumin-induced constriction, there was still an appreciable bronchoprotective effect of Cmpd-6 against methacholine-induced airway constriction — particularly in human lung slices — suggesting that PAMs of the β_2_AR will benefit patients with asthma irrespective of the stimulus that causes their airway constriction.

We were unsuccessful at detecting any effect of Cmpd-6 on albuterol-induced bronchoprotection in mice in vivo. To explain this lack of effect we found that F-133, a crucial amino acid for Cmpd-6 binding in the human β_2_AR (22), is replaced by V-133 in the murine β_2_AR, unlike the guinea pig receptor where F-133 is conserved. Substitution of the nonconserved murine amino acid with its human counterpart completely rescued the PAM activity of Cmpd-6 in the murine β_2_AR, in vitro. The fact that Cmpd-6 lacks the proper allosteric binding site on the murine β_2_AR and does not potentiate β_2_-agonist–mediated bronchodilation in mice demonstrates that the enhanced bronchoprotection observed in human and guinea pig lung slices is specific to the β_2_AR. Our findings regarding Cmpd-6 species–specificity toward the β_2_AR are consistent with previous studies that showed species-dependent PAM activities toward other GPCRs (51–53). Since allosteric sites generally exhibit a greater variation between species than the orthosteric sites (54, 55), species-dependent effects may be even more prevalent for allosteric modulators. During the allosteric drug development process, species-specific differences would need to be considered not only in screening efforts (25, 56), but also in selection of appropriate animal models that relate to the pathophysiology and pharmacology of the human disease. Our studies further indicate the importance of determining the location of allosteric binding sites by structural approaches.

In conclusion, we demonstrate that the β_2_AR-selective PAM, Cmpd-6, enhances the bronchoprotective effect of the β_2_-agonist fenoterol against methacholine-induced airway narrowing in guinea pig and human lung slices as well as against allergen-induced airway narrowing in guinea pig lung slices. Our study suggests that PAMs like Cmpd-6 hold promise as adjuncts to β_2_-agonists to improve control of airway narrowing in asthma. Given the exceptional β_2_AR selectivity and ceiling effect of PAMs, Cmpd-6 may improve the pharmacological treatment of asthma and other respiratory diseases by increasing the bronchoprotective response to β_2_-agonists, lowering the effective dose of β_2_-agonists, and producing fewer side effects.

## Methods

### Materials.

Compound-6 was synthesized as previously described (21). With the exception of BI-167107, which was synthesized as previously described (57), all of the orthosteric β_2_AR ligands used, methacholine, and ovalbumin were purchased from Sigma-Aldrich and sourced at a 95% or greater purity. All other chemicals were obtained from Sigma-Aldrich unless otherwise indicated. Mammalian expression plasmids for human and murine β_2_ARs were previously described (36) and obtained from GeneScript. The plasmid for the guinea pig β_2_AR was generated by insertion of the de novo synthesized coding region of the receptor into pcDNA3 through 5′-EcoR1 and 3′-NotI sites by GENewIZ. The V133F mutant of the murine β_2_AR was created using the Quikchange Site-Directed Mutagenesis Kit (Agilent). The V133F mutation was verified by sequencing. The coding regions of all plasmids were also sequenced for their authentication.

### Cell culture and transfection.

Human embryonic kidney-293 (HEK293) cells (ATCC) stably expressing the GloSensor cAMP reporter (58) and HEK293T cells for the Tango assay with the β_2_V_2_R chimeric receptor (59) were maintained at 37°C and 5% CO_2_ in a humidified condition. Cells were cultured in standard MEM (Gibco) supplemented with 10% FBS and penicillin/streptomycin (GeminiBio) together with proper selection antibiotics, 100 μg/mL Hygromycin B (Invitrogen) for the GloSensor cells and 300 μg/mL Zeocin (Invitrogen), 100 μg/mL Hygromycin B, and 5 μg/mL of puromycin (Gibco) for the Tango cells. Expi293F suspension cells (Invitrogen) were maintained in Expi293 Expression Medium (Thermo Fisher Scientific) at 37°C and 8% CO_2_ in a humidified condition while shaking the culture flask. Each construct for expression of the β_2_AR from different species was transiently transfected into HEK293 GloSensor cells using FuGENE 6 (Promega) for cAMP production measurement and Expi293F cells using Expifectamine (Invitrogen) for membrane isolation according to the manufacturer’s instructions. All the assays and preparations were performed at approximately 48 hours after transfection.

### Measurements of cAMP production.

HEK293 cells stably expressing the GloSensor luciferase enzyme (Promega), in the absence or presence of transient transfection with each of the constructs for expressing the β_2_AR from different species, were plated at a density of approximately 80,000 cells on each well of a 96-well, white clear-bottom plate. At 20–24 hours after plating the cells, the GloSensor reagent (Promega) was prepared and added onto the cells in each well according to the manufacturer’s instructions. The plate was moved to a humidified incubator at 27°C, and after 1 hour incubation, cells were then treated with Cmpd-6 at an indicated concentration or a vehicle (DMSO) diluted in HBSS (Sigma-Aldrich), supplemented with 20 mM Hepes, pH 7.4, 0.05% BSA, and 100 μM 3-isobutyl-1-methylxanthine (IBMX) (Sigma-Aldrich). After cells were further incubated for 20 minutes, the extent of luminescence signal was read using a ClarioStar microplate reader (BMG Labtech). When endogenously expressed β_2_AR in HEK293 cells was stimulated with a serial dilution of a β_2_ agonist, a luminescence reading was performed 10 minutes after stimulation.

### Measurement of β-arrestin recruitment.

β-Arrestin2 recruitment to the receptor was measured using the previously described Tango assay (59). HEK293T cells stably expressing the β_2_V_2_R tethered to the tetracycline transactivator (tTA) transcription factor with a Tobacco Etch Virus (TEV) protease cleavage site as a linker. The β_2_V_2_R is a chimeric receptor created by fusion of the C-terminal region of the V2 vasopressin receptor to the C-terminal-truncated β_2_AR to improve the extent of β-arrestin recruitment to the receptor while retaining the pharmacological profile of the WT β_2_AR (35). These Tango cells are also stably express human β-arrestin2 fused to the TEV protease and the tTA-driven luciferase reporter. At 20–24 hours after cells were plated on a 96-well, white clear-bottom plate at a density of approximately 50,000 cells per well, they were treated with either Cmpd-6 at 25 μM or a vehicle (DMSO) diluted in HBSS (Sigma-Aldrich), supplemented with 20 mM Hepes, pH 7.4, and 0.05% BSA. After 20 minutes incubation at 37°C, cells were stimulated with a serial dilution of a β_2_ agonist, followed by 6 hours further incubation at 37°C in a humidified condition. Then, after the plate was briefly cooled down to the ambient temperature, the Bright-Glo reagent (Promega) was added following the manufacturer’s instructions to read chemiluminescence signals using a ClarioStar microplate reader (BMG Labtech).

### Radioligand Competition Binding.

Plasma membranes from the Expi293F cells (Thermo Fisher Scientific) transiently expressing each of the WT or mutant receptors were prepared as described previously (60). With each of the prepared membranes, radioligand competition binding assays were performed using a radiolabeled antagonist, [^125^I]-cyanopindolol (CYP) (2,200 Ci/mmol; PerkinElmer) at a concentration of 60 pM. Reactions started upon mixing the isolated membranes together with ^125^I-CYP, Cmpd-6 at varying concentrations, and a serial dilution of a competitor β_2_-agonist in an assay buffer (75 mM Tris-HCl, pH 7.4, 2 mM EDTA, pH 8.0, 12.5 mM MgCl_2_, 0.1% BSA, and 1 mM ascorbic acid), as indicated on each figure. Reaction mixtures were incubated for 90 minutes at ambient temperature to reach the equilibrium state. Assays were then terminated by rapid filtration of the reaction mixtures onto GF/B glass-fiber filters (Brandel) treated with 0.3% polyethyleneimine and washed with 8 mL of a cold binding buffer (75 mM Tris-HCl, pH 7.4, 2 mM EDTA, pH 8.0, 12.5 mM MgCl_2_) using a harvester (Brandel). The extent of ^125^I-CYP bound to the β_2_AR in isolated membranes was measured using a WIZARD^2^ 2-Detector Gamma Counter (PerkinElmer). Data were expressed as specific binding obtained by subtraction of nonspecific binding determined in the presence of high-affinity propranolol at 20 μM.

### Mice.

Seven-to-14-week-old male C57BL/6J naive mice (*n* = 5–7) were purchased from the Jackson Laboratory and were housed in pathogen-free temperature- and humidity-controlled facilities at Duke University. Mice were euthanized at the end of airway responsiveness measurement.

### Airway responsiveness measurements in vivo.

Airway hyperresponsiveness (AHR) was measured using the forced oscillation technique as described previously (42). In brief, anesthetized (sodium pentobarbital 85 mg/kg, i.p.), tracheotomized, and skeletal muscle-relaxed (pancuronium bromide 0.25 mg/kg, i.v.) mice were i.v. administered with Cmpd-6 (50 mM in 100% DMSO; 10 mg/kg), or equivalent volume of 100% DMSO as vehicle control for 10 minutes before the start of the bronchoprotection or bronchodilation protocol as described below. For the bronchoprotection protocol, bronchospasm was induced by jugular vein i.v. administration of increasing doses of methacholine (25, 50, 100, 200, 400 μg/kg). For the bronchodilation protocol, a consistent response to i.v. 125 μg/kg methacholine was established immediately prior to administration of 125 μg/kg methacholine combined with increasing doses of albuterol (1, 3, 10, and 30 μg/kg). Mice were ventilated with 100% oxygen at 150 breaths per minute, constant volume of 8 mL/kg and a 3 cm H_2_O positive end–expiratory pressure using a computer-controlled small animal ventilator (flexiVent, SCIREQ). The total lung impedance signal collected contained information about the resistance and elastance properties of the lung from which Newtonian resistance (R_n_), a good indicator of the luminal diameter of the conducting airways, was calculated using the constant phase model. The heart rate data acquired using EKG electrodes was monitored to assess mouse viability and analgesia throughout the bronchoconstrictor protocols.

### Guinea pig model of allergic asthma.

Outbred, male, specified pathogen-free Dunkin Hartley guinea pigs (Charles River Laboratory) weighing 200 to 400 g were used. The animals were housed in pairs under a 12-hour light/dark cycle in a temperature- and humidity-controlled room with food and tap water ad libitum. For studies in the guinea pig model of allergic asthma, animals were actively IgE-sensitized to ovalbumin by injecting a suspension containing 100 μg/mL ovalbumin and 100 mg/mL Al(OH)_3_ in saline. Each animal was injected with 1.0 mL of suspension: 0.5 mL was injected *i.p*. and 0.5 mL was divided over 7 s.c. sites close to lymph nodes in the neck, paws, and lumbar regions (24). Animals were euthanized at least 4 weeks after sensitization.

### Guinea pig lung slices.

Precision-cut lung slices were prepared as described previously (61, 62). A 3% solution of low melting-point agarose (Gerbu Biotechnik GmbH) in ultrapure water was prepared by heating it a microwave until fully dissolved. A double concentrated lung slice buffer (3.6 mM CaCl_2_, 1.6 mM MgSO_4_, 10.8 mM KCl, 232.8 mM NaCl, 2.4 mM NaH_2_PO_4_ (Thermo Fisher Scientific), 33.4 mM glucose (Spectrum Chemicals), 52.2 mM NaHCO_3_, 50.4 mM Hepes, pH = 7.2) was 1:1 mixed with the 3% agarose solution after bringing both at 37°C. Animals were sacrificed using an overdose of pentobarbital (Euthasol, Patterson Veterinary) followed by exsanguination via the aorta abdominalis. Lungs were filled through a tracheal cannula at constant pressure with a 1.5% low melting-point agarose solution in lung slice buffer at 37°C with 1 μM isoproterenol added to prevent postmortem airway constriction. After filling, the lungs were covered with ice for 30 minutes to solidify the agarose for slicing. Lungs were removed and cylindrical tissue cores (diameter 15 mm) were prepared followed by slicing the tissue in ice cold lung slice buffer (1.8 mM CaCl_2_, 0.8 mM MgSO_4_, 5.4 mM KCl, 116.4 mM NaCl, 1.2 mM NaH_2_PO_4_, 16.7 mM glucose, 26.1 mM NaHCO_3_, 25.2 mM Hepes, pH = 7.2) using a tissue slicer (CompresstomeTM VF- 300 microtome, Precisionary Instruments). Lung slices were cut at a thickness of 500 μm and washed several times with slicing buffer to remove debris and washout the isoproterenol. Slices were incubated overnight in a 60 mm dish in sterile incubation buffer: MEM composed of lung slice buffer supplemented with 0.5 mM sodium pyruvate, 1 mM glutamine (Gibco), MEM-amino acids mixture (1:50), MEM-vitamins mixture (1:100; Gibco), and penicillin-streptomycin (1:100; Gibco), pH = 7.2, at 37°C in a CO_2_- and humidity-controlled atmosphere.

After washing the slices in medium, individual guinea pig lung slices were mechanically maintained with a Teflon ring with an inner diameter of 7 mm and covered with 1 mL of incubation buffer. Airway responsiveness to increasing concentrations of methacholine (10 nM to 3 mM, using cumulative concentrations in half-log increments) or ovalbumin (1 pg/mL to 1 mg/mL, using cumulative concentrations in log increments) was measured in lung slices from naive or IgE-sensitized guinea pigs, respectively, using video-assisted microscopy (Nikon Eclipse TS 100) as previously described (61). Lungs slices were incubated with various concentrations of the β_2_-agonist fenoterol (0.1, 1, 10, and 100 μM) and/or Cmpd-6 (25 μM) before the addition of methacholine or ovalbumin. Image acquisition software (NIS-Elements, Nikon) was used to quantify airway luminal area. Images of the airways were acquired every 2 seconds during the whole course of the experiment, starting 2 minutes before the addition of any agent to allow for baseline measurements of the airway caliber. For each methacholine or ovalbumin concentration, the maximal airway constriction was expressed as percentage of the initial (baseline) airway luminal area and plotted against that concentration. The maximal constriction (E_max_, percentage airway closure) and concentration of methacholine or ovalbumin inducing 50% of the maximal response (EC_50_) were determined for each concentration-response curve.

### Human lung slices.

Precision-cut human lung slices were purchased from AnaBios and stored in a liquid nitrogen tank. Lung tissue was obtained postmortem and consent for research was obtained by AnaBios for all donors. Characteristics of the donors of the lung tissue are shown in Supplemental Table 2. Slices were rapidly thawed following instructions from AnaBios. In short, slices were washed 3 times in DMEM/F12 medium supplemented with penicillin-streptomycin (1:100) followed by a wash in incubation buffer (1.8 mM CaCl_2_, 0.8 mM MgSO_4_, 5.4 mM KCl, 116.4 mM NaCl, 1.2 mM NaH_2_PO_4_, 16.7 mM glucose, 26.1 mM NaHCO_3_, 25.2 mM Hepes, 0.5 mM sodium pyruvate, 1 mM glutamine, MEM-amino acids mixture (1:50), MEM-vitamins mixture (1:100) and penicillin-streptomycin (1:100), pH = 7.2) and subsequently placed overnight in incubation buffer at 37°C in a CO_2_- and humidity-controlled atmosphere.

After washing, individual human lung slices were mechanically maintained with a Teflon ring with an inner diameter of 7 mm and covered with 1 mL of incubation buffer. Airway responsiveness to increasing concentrations of methacholine (1 nM to 300 μM, using cumulative concentrations in half-log increments) was measured using video-assisted microscopy (Nikon Eclipse TS 100). Lungs slices were incubated with the β_2_-agonist fenoterol (1 and 10 μM) for 30 minutes before the addition of methacholine. For the condition where fenoterol and Cmpd-6 were used, lung slices were incubated with 25 μM Cmpd-6 5 minutes before the addition of 1 μM fenoterol. Image acquisition software (NIS-Elements, Nikon) was used to quantify airway luminal area. The initial airway intraluminal area was assessed after fenoterol incubation and before methacholine incubation. Airway constriction in response to each methacholine concentration was expressed as percentage of the initial airway luminal area. The maximal constriction (E_max_, percentage airway closure) to methacholine and –log of the concentration of methacholine inducing 50% of the maximal response (pD_2_) were determined for each concentration-response curve.

### Statistics.

Statistical differences were determined using a 1-way ANOVA followed by either Bonferroni’s (Tables 2 and 3) or Tukey’s (Figure 3D) posthoc test. General linear model repeated measures ANOVA with Tukey’s posthoc test was used to determine differences in airway responsiveness in vivo (Figure 2). Differences were considered to be statistically significant when *P* < 0.05. All other analyses were performed using paired 2-tailed Student’s *t* tests. All curve fits were generated using the software program GraphPad Prism.

### Study approval.

All animal care and experimental procedures complied with the animal protection and welfare guidelines and were approved by the Institutional Animal Care and Use Committee of Palm Beach Atlantic University or Duke University and are reported in compliance with the ARRIVE guidelines (63).

### Data availability.

Data are available in the Supporting Data Values XLS file. All data needed to evaluate the conclusions of this study are available in the paper. Cells for the Tango β-arrestin recruitment assay were a gift from Gilad Barnea (Brown University, Providence, Rhode Island, USA). Cells for the GloSensor cAMP production assay can be obtained upon request to RJL and used for academic research with a standard academic material transfer agreement (MTA).

## Author contributions

SA, HM, JKLW, SL, AH, and RJL conceived and designed experiments. SA, HM, JKLW, SL, AH, and HCS performed experiments and analyzed the data. AWK synthesized the allosteric compound (Cmpd-6). SA, HM, JKLW, and RJL wrote the manuscript. All authors contributed feedback to the manuscript and approved the submitted version. SA, HM, and JKLW share the first author position. The alphabetical order of their last names was used to assign the authorship order among these authors.

## Supplementary Material

Supplemental data

Supporting data values

## Figures and Tables

**Table 3:**
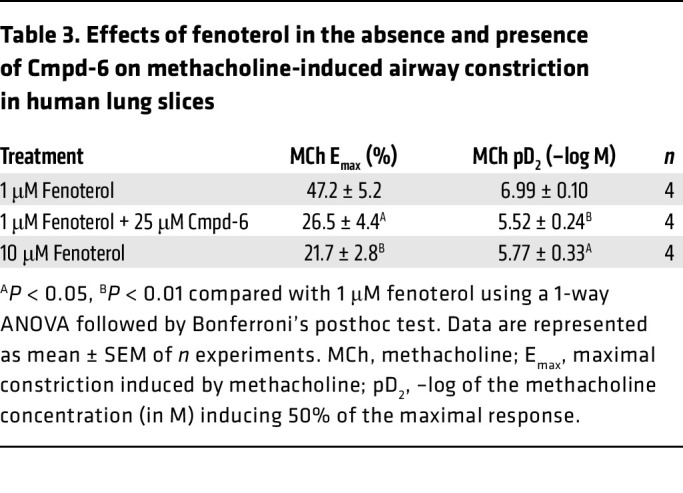
Effects of fenoterol in the absence and presence of Cmpd-6 on methacholine-induced airway constriction in human lung slices

**Table 2:**
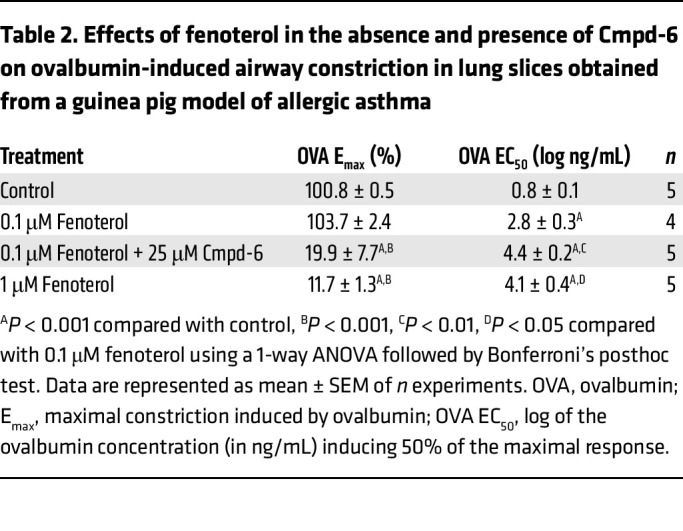
Effects of fenoterol in the absence and presence of Cmpd-6 on ovalbumin-induced airway constriction in lung slices obtained from a guinea pig model of allergic asthma

**Table 1:**
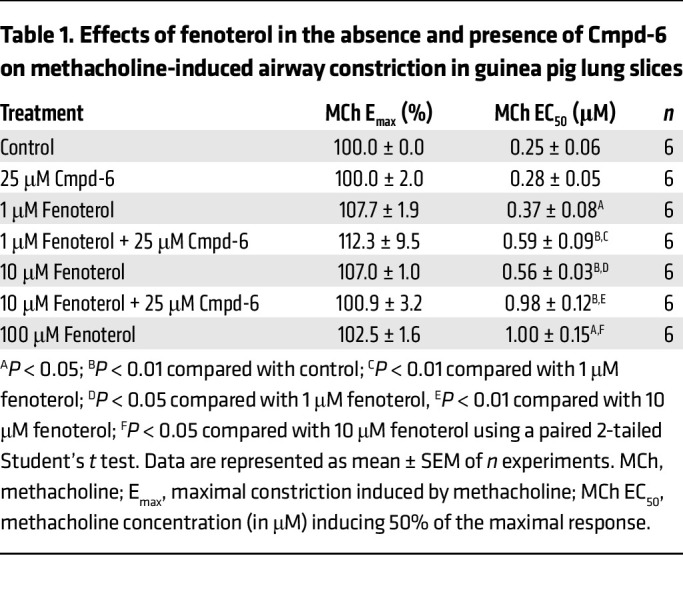
Effects of fenoterol in the absence and presence of Cmpd-6 on methacholine-induced airway constriction in guinea pig lung slices

## Version 1 07/11/2023

In-Press Preview

## Version 2 09/15/2023

Electronic publication
