# Supplementary material for: Allosteric modulator potentiates β2AR agonist–promoted bronchoprotection in asthma models
Source: J Clin Invest. 2023 Sep 15;133(18):e167337. doi: 10.1172/JCI167337 (PMC10503797; doi:10.1172/JCI167337)
Supplement: Supplemental data [file jci-133-167337-s079.pdf]

Supplemental Table 1 – Cmpd-6 physicochemical characteristics.

**Structure**	**Molecular Weight**		612
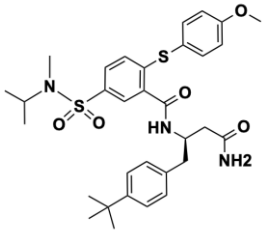	**Solubility***	DMSO	100 mM
		Aqueous	32 µM
	**Cell Permeability****		YES
	**Log P*****		5.69

*Solubility was determined by formation of precipitates observed under the microscope. **Cell permeability was determined by the functional activity of Cmpd-6 in cell-based assay since it binds an intracellular region of the β_2AR. ***Log P, Log of the Partition coefficient that was calculated using ChemDraw Professional (version 22.2, PerkinElmer). DMSO, dimethyl sulfoxide.

Supplemental Table 2 – Donor characteristics for the human lung slices.

**Treatment**	**Donor 1**	**Donor 2**	**Donor 3**	**Donor 4**
Age	45	47	14	35
Sex	M	F	F	F
Ethnicity	Caucasian	Caucasian	African-American	Caucasian
BMI	29.8	24.6	23.7	24.0
History of respiratory disease	No	No	No	Yes[1]
Cause of death	Head Injury/ Blunt Trauma	CVA/ICH/Stroke	Anoxia/Drug Intoxication	Anoxia/Natural Causes

[1] Acute resp failure with hypoxia, *Klebsiella* pneumonia infection, acute infective tracheobronchitis.
